# The serotonin 1A receptor C(-1019)G polymorphism in relation to suicide attempt

**DOI:** 10.1186/1744-9081-2-14

**Published:** 2006-04-20

**Authors:** Danuta Wasserman, Thomas Geijer, Marcus Sokolowski, Vsevolod Rozanov, Jerzy Wasserman

**Affiliations:** 1 The National and the Stockholm County Centre for Suicide Research and Prevention of Mental Ill-Health (NASP), Dep. of the Public Health Sciences, Karolinska Institute, Stockholm, Sweden; 2 The Human Ecological Health Organization/Odessa National Mechnikov University, Odessa, Ukraine

## Abstract

**Background:**

Serotonergic neurotransmission has been implicated in suicidal behavior. Association between suicidal completers and a regulatory C(-1019)G polymorphism (rs6295) in the serotonin 1A receptor (HTR1A) gene was previously reported, whereas a following study showed no association in a sample of suicide attempters.

**Methods:**

The involvement of the implicated G-allele of the 5-HTR1A C(-1019)G polymorphism (rs6295) was analyzed with the transmission disequilibrium test (TDT) in a sample of 272 suicide attempter families.

**Results:**

No overtransmission of the G-allele was found in the entire sample of suicide attempters (p = 0.1460; n = 272 trios). However, a strong trend for overtransmission of the G-allele was observed in a sub-sample selected for a high level of previous traumatic and/or stressful life events prior to the suicide attempt (p = 0.0630, two-tail; n = 94 trios).

**Conclusion:**

The current results show that variation at the rs6295 polymorphism of the HTR1A gene is not associated with suicide attempts generally. However, the results indicate a possible role of the G-allele in suicidal behavior in connection with high exposure to traumatic and/or stressful life events, which is in need of future investigation.

## Background

During the last three decades, evidence indicating that altered serotonergic neurotransmission influences suicidal behavior has accumulated. One of the more consistent findings is the observation of low cerebrospinal fluid (CSF) levels of 5HIAA (5-hydroxy-indole-acetic-acid, a serotonin metabolite) among suicide attempters [1-3]. As a consequence, research regarding specific genes that may influence suicidal behavior has mostly, focused on the serotonergic system [4].

In studies focused on the serotonin 1A receptor (HTR1A) gene, Nishiguchi et al. [5] found no association between suicide and two structural polymorphisms, Pro16Leu and Gly272Asp in this gene. However, a later study [6] observed a relationship between suicide and a functional single nucleotide polymorphism (SNP), C(-1019)G (rs6295), that appears to be directly involved in the regulation of transcription of the HTR1A gene. In a series of experiments, it was shown that the part of the HTR1A gene promoter that contains SNP rs6295 binds to the transcription factor named nuclear deformed epidermal autoregulatory factor DEAF-1/suppressin (NUDR), that this factor represses transcription of HTR1A and that this inhibitory action was impaired for the G-allele, the same allele that was associated with suicide and major depression [6]. The implication is that failed inhibition by NUDR at the G-allele leads to higher expression of HTR1A. This could enhance the negative feedback inhibition of serotonergic raphe neurons exerted by HTR1A autoreceptors and lead to a lower serotonergic neurotransmission, which is consistent with the observation of low serotonin-levels in depression and suicide. However, a more recent study of rs6295 by Huang et. al [7] showed no association with the G-allele among suicide attempters in relation to healthy volunteers. Furthermore, the most recent report showed no association of the G-allele to alcoholism and suicide attempts generally, but association to Babors type B alcoholism and number of suicide attempts in history [8]. The discrepancies between these studies may reflect a problem of population stratification, which can be overcome by using the transmission disequilibrium test (TDT) [9].

In the present study, the indicated involvement of the G-allele of SNP rs6295 in suicidal behavior is tested with the TDT in a sample of 272 suicide attempter families.

## Methods

### Research subjects

Triplets (proband and both parents) were collected as previously described [10]. The collection of research subjects followed the code of ethics of the World Medical Association (Declaration of Helsinki). Before participation in the study, all research subjects were given oral and written information about the project. A written consent was subsequently obtained from them. The study was approved by the Research Ethics Committee at the Karolinska Institutet (Dnr 97–188), and by the Ministry of Health in Ukraine. The triplet sample consisted of 272 triplets. Inconsistent genotype pattern within families had been investigated before triplets were included in the sample. All research subjects were Caucasian, and Ukrainian citizens. Of the probands, 61.0 % had at least three grandparents of Ukrainian nationality, 33.1 % had at least three grandparents who were of Ukrainian or Russian nationality and 5.2 % had two, or more, grandparents of other origin than Ukrainian or Russian, predominantly from other East-European regions. The average ages of the 272 probands were 24.3 ± 7.5 years. The female/male rations among probands were 45.2%/54.8%. ICD-10 diagnoses were determined using a computerized version of the Composite International Diagnostic Interview (CIDI), core version 2.1, and adjustment disorder diagnosis according to DSM IV criteria. Among probands, mild depressive episodes were present among 3.3%. Moderate depressive episodes were present among 4.4% and severe depressive episodes among 5.9%. Dysthymia was present among 6.6%. Panic disorder, phobias obsessive compulsive disorder, posttraumatic stress, or generalized anxiety disorder were present among 29.8%. Schizophrenia, delusional disorder or acute and transient psychotic disorders were present among 5.9%. Harmful substance use or dependence syndromes were present among 19.1% and bipolar disorder was diagnosed for 0.7%. Adjustment disorder was observed for 13.2%. Previous suicide attempts were admitted by 30.5%.

### Medical damage rating score (MDS)

The lethality of the index suicide attempt was evaluated with the help of the Medical Damage Rating Scale [11], scoring medical damage from a suicide attempt from 0 (none) to 8 (dead). A medical damage score ≥ 2 for the proband was required for inclusion. A damage score ≥ 4 for the proband was used to select the "MDS high" sample of triplets.

### Life events (LE)

An experimental Life Event Inventory (LEI) was used to measure the amount of traumatic life events among research subjects. Two versions were developed. The first version (LEI-1) consisted of 69 items, questions corresponding to various traumatic and/or stressful events across the life span (involving e.g. accidents, injuries, illness, attacks, threats, loss, rape/sexual abuse, torture, poverty, homelessness, defeat, loneliness, humiliation, relational conflicts), of which 46 items were used for scoring. The items were selected from the Composite International Diagnostic Interview (CIDI), core version 2.1, section K, and from the interview-schedule (the European Parasuicide Study Interview Schedule) used in the WHO/EURO Multicentre Study on Parasuicide [12]. In a developed version (LEI-2), the questionnaire was revised: the number of items was reduced to 28, and the item-content, in most cases, modified into a more general form. For both versions, items were scored and grouped as follows: a score above the 75th percentile was used to designate probands as "life events high" (LE high) and a score below the 25th percentile as "life events low" (LE low). The percentiles used were obtained from the skewed LE-distribution resulting from interviewing 334 healthy volunteers. Both LEI instruments were alternately used in the present project. The LEI-1 was used in early phases of the collection of triplets while the LEI-2 was used in a later phase. Although the later version was revised, a good correlation in designating LE high vs LE low remained between both versions, as demonstrated by interviewing 50 general population subjects using both versions cross-wise, with a one-week lapse between interviews (p = 0.034, Fishers exact test).

### DNA preparation

Venous blood (10 ml) was taken from all research subjects into EDTA-containing tubes. DNA isolation was, with slight modifications, performed as previously described [13].

### Genotyping

Genotyping of rs6295 was performed by the custom genetic analysis services using the high throughput BeadArray™ technology at Illumina, Inc., CA.

### Transmission disequilibrium test (TDT) analysis

Relationships between the genetic markers and suicide attempt were investigated by means of TDT analysis [9] using software developed within the Genetic Investigation of Suicide attempt and Suicide (GISS)-project. Statistical power was calculated using the software *TDT Power calculator *[14].

## Results

TDT-analysis was performed for the rs6295 polymorphism using genotype-data from 272 triplets. The analysis performed was two-tailed, since the previously reported implication of the G-allele in suicidal behavior by Lemonde et al. [6], could not be replicated in a following study by Huang et. al [7]. The frequency of the rs6295 G-allele was 54% in the present sample. With that frequency and at a significance level of 5%, a power of 85% was obtained for a dominant model, hypothesizing the penetrances 0.045, 0.045 and 0.015 for the genotypes GG, GC and CC, respectively. The penetrances were chosen to match the life time prevalence for suicide attempt, which has been estimated to 3–5% [15-17].

There was no significant difference in transmission frequency when the entire sample was analyzed (p = 0.1460), displaying a weak tendency in the expected direction (Table 1). Because low CSF levels of 5HIAA have been correlated with high levels of lethality at the suicide attempt [1], a sub-sample (MDS high) of triplets was selected, wherein all probands had suffered high medical damage. This may also bring the suicide attempter closer to resemble a suicide completer, which is more comparable to the sample which was previously shown to have a higher prevalence for the G-allele [6]. The analysis of the MDS high sample here showed no significant difference in transmission of the G-allele (p = 0.1911), similar to the results obtained with the entire sample (Table 1).

**Table 1:** Transmission Disequilibrium Test (TDT) Analysis (Two-Tailed) of Single Nucleotide Polymorphism (SNP) rs6295 in Suicide Attempt

Sample	Triplets	Transmission G-allele	Transmission C-allele	*P*-value
Total	272	148	124	0.1460
MDS high	107	64	50	0.1911
LE high	94	56	38	0.0636
LE low	60	31	30	0.9587

Genetic variation in another serotonergic component, the serotonin transporter (SERT), have previously been shown to interact with stressful life events to promote suicide attempts [18]. Thus, when an interaction exists between life events and the genetic component, which act together to promote suicidality, the effect may be expected to increase in a sub-sample enriched for high levels of life events. Therefore, a LE high sample was analyzed, in which the attempters scored high on traumatic and/or stressful events across the life span. To contrast this, another sub-sample (LE low) was also analyzed, in which the selection was based on a low level of traumatic and/or stressful life events. Had the genetic component not been dependent on interaction with life events, and instead related more directly and independently with the suicidal behavior *per se*, such an effect would be more likely to be detected in a sample with minimized levels of life events. Whereas the analysis of the LE low sample showed no significant transmission of the G-allele (p = 0.9587), a strong trend for overtransmission of the G-allele was evident in the LE high sample (p = 0.0636) (Table 1). At a significance level of 5%, and increasing the penetrances (compared with the entire sample) to 0.05, 0.05 and 0.01 for genotypes GG, GC and CC, respectively, power was calculated to 66%, 59% and 40% for sub-samples MDS high, LE high and LE low, respectively

Taken together, we could not detect significant overtransmission of the G-allele, in neither the total sample, nor sub-samples MDS high, LE low or LE high. However, we note the presence of a strong tendency for overtransmission of the G-allele in the LE high sample.

## Discussion

Whereas the present investigation does not provide evidence for the involvement of the G-allele of SNP rs6295 in suicide attempt generally, the data gives an indication of a possible role of the G-allele in suicide attempters exposed to high levels of traumatic and/or stressful life events.

We chose TDT as the method for analysis since it is a test for both linkage and association, enhancing the possibility to draw general conclusions. It also reduces the effect of population stratification which in case/control designs may cause spurious associations. However, the G-allele frequency in the Ukrainian sample was 54 % which should be compared to the life-time prevalence of suicide attempt of 3–5%. The statistical power for TDT analysis is highly decreased in such conditions. An rs6295 G-allele frequency more similar to what was observed in the sample analyzed by Lemonde et al. (i.e. 15%), in which completed suicide was associated with the G-allele [6], would have offered a better opportunity for observing a potential effect. In this view, our sample is more similar to that studied by Huang et. al [7], which used suicide attempters with a G-allele frequency of 53%. Our results are also consistent with the results reported by Huang et. al [7], in that there is no increased prevalence of the G-allele among suicide attempters generally.

An allele frequency of about 50%, when life-time prevalence of suicide attempters is about 4%, also implicates a moderate/general effect and low penetrance. In our investigation we had to hypothesize a genotype-risk of 2.5 for the total sample to reach a power of > 80%. Furthermore, a genotype-risk of 5.0 hypothesized for the sub-samples, resulted in powers of < 80%. More reasonable genotype-risks are likely in the range of 1.5 to 2.0. A genotype risk of 1.5 would require a sample of close to 900 trios. However, because of the lack of statistical power in the sub-sample analyses, the results obtained with the LE high sample may interpreted as a possible trend for association and transmission disequilibrium of the G-allele. We speculate that if one would increase the statistical power to 80% for the sub-samples, this would indicate the G-allele in the LE-high sample but not in MDS high or LE low.

We further speculate that if the TDT-analyses were to be considered as one-tailed, the results in the LE high sub-sample would be significant (p < 0.05). In fact, the present study was initially designed as an attempt to replicate the results reported by Lemonde et al. [6]. A positive result in the LE high sample would be consistent with the results obtained for another serotonergic component, SERT, i.e. that higher levels of stress increase the genetic effects in suicidal behavior [18].

Interestingly, the SERT-polymorphism is further comparable to rs6295, in that both result in a regulatory effect on gene expression. Our results in the LE high group, which indicated a possible trend among suicide attempters, may be viewed as being in contrast with those reported by Huang et al. of no association among suicide attempters generally [7], whereas being consistent with those reported by Lemonde et al., demonstrating association among suicide completers and major depression [6]. An explanation of these discrepancies may be that the phenotype associated with the rs6295 polymorphism, is still not optimally defined (e.g. suicidal behavior being a heterogonous phenotype). Instead, our LE high sample may have certain similarities with all the samples indicated in previous reports. Huang et al. demonstrated an association with the clinical characteristics of schizophrenia, substance use disorder and panic attack [7]. Interestingly, this may be compared to the sub-samples studied here, where the similar clinical characteristics were present in higher proportions in LE high (43.6%), compared to LE low (16.7%) and MDS high (10.9%), respectively. The first obvious similarity with the report by Lemonde et al. is suicidal behavior. Lemonde et al. also reported of association with major depression [6]. This may also be compared to the sub-samples studied here, where moderate/major depression was present in higher proportions in LE high (23.4%), compared to LE low (6.7%) and MDS high (6.2%), respectively. Finally, Koller et al. recently found no association of the G-allele to alcoholism and suicide attempts generally, but association to Babors type B alcoholism and the number of suicide attempts in history [8]. Interestingly, Babors type B alcoholism is typified by e.g. more life stress. Thus, besides suicidal behavior, the LE high sample indicated here showed a higher aggregation of many of the characteristics shown to be associated with the G-allele in previous reports. Conclusively, this further indicates that the results presented here with the LE high sub-sample, may constitute a promising and consistent trend. A more detailed definition of the rs6295-phenotype in the combined relation between suicidal behavior, clinical characteristics and life events is likely to be more informative in future studies.

## Conclusion

Taken together, a general effect on suicide attempt attributed to the rs6295 G-allele, does appear not to be the case. However, since the analysis of the LE high sample showed a promising trend, we consider the results in need of replication in a sample with greater statistical power.

## Competing interests

The author(s) declare that they have no competing interests.

## Authors' contributions

D.W., T.G. and J.W. participated in the design of the study, the statistical analysis and in the writing of the manuscript. M.S. performed the statistical analysis and participated in the writing of the manuscript. V.R. participated in the coordination and collection of the sample material.
